# PTEN/PTENP1: ‘Regulating the regulator of RTK-dependent PI3K/Akt signalling’, new targets for cancer therapy

**DOI:** 10.1186/s12943-018-0803-3

**Published:** 2018-02-19

**Authors:** Nahal Haddadi, Yiguang Lin, Glena Travis, Ann M. Simpson, Eileen M. McGowan, Najah T. Nassif

**Affiliations:** 1 School of Life Sciences, Faculty of Science, University of Technology Sydney, 15 Broadway, Ultimo, Sydney, NSW 2007 Australia; 2 Central Laboratory, The First Affiliated Hospital of Guangdong Pharmaceutical University, Guangzhou, 510080 China

**Keywords:** Phosphatase and tensin homologue (PTEN), PTENP1, Pseudogene, Tyrosine kinase, PI-3 kinase (PI3K), Cancer, microRNA (miRNA)

## Abstract

Regulation of the PI-3 kinase (PI3K)/Akt signalling pathway is essential for maintaining the integrity of fundamental cellular processes, cell growth, survival, death and metabolism, and dysregulation of this pathway is implicated in the development and progression of cancers. Receptor tyrosine kinases (RTKs) are major upstream regulators of PI3K/Akt signalling. The phosphatase and tensin homologue (PTEN), a well characterised tumour suppressor, is a prime antagonist of PI3K and therefore a negative regulator of this pathway. Loss or inactivation of PTEN, which occurs in many tumour types, leads to overactivation of RTK/PI3K/Akt signalling driving tumourigenesis. Cellular PTEN levels are tightly regulated by a number of transcriptional, post-transcriptional and post-translational regulatory mechanisms. Of particular interest, transcription of the PTEN pseudogene, PTENP1, produces sense and antisense transcripts that exhibit post-transcriptional and transcriptional modulation of PTEN expression respectively. These additional levels of regulatory complexity governing PTEN expression add to the overall intricacies of the regulation of RTK/PI-3 K/Akt signalling. This review will discuss the regulation of oncogenic PI3K signalling by PTEN (the regulator) with a focus on the modulatory effects of the sense and antisense transcripts of PTENP1 on PTEN expression, and will further explore the potential for new therapeutic opportunities in cancer treatment.

## Background

The phosphatase and tensin homologue (PTEN) is essential for normal cell maintenance and is well characterised as a key tumour suppressor [1]. PTEN is pivotal in the regulation of the receptor tyrosine kinase (RTK) PI-3 kinase (PI3K)/Akt signalling pathway and, as such, even small changes in PTEN expression have been shown to have major consequences for normal cellular function [2–5]. The PTEN protein translocates between the nucleus and the cytoplasm enabling PTEN-specific compartmentalised functions [6, 7]. At the molecular level, PTEN expression and cellular abundance is tightly regulated at the transcriptional, post-translational and post-transcriptional levels. In recent years, there has been much interest in the PTEN pseudogene (PTENP1) as a novel negative and positive modulator of PTEN expression.

The PI3K/Akt pathway is activated subsequent to RTK activation. Hyperactivation of PI3K/Akt signalling has been reported in many types of human cancers, thus targeting the regulators in this pathway has attractive therapeutic potential. As such, a large number RTKs and PI3K candidates are under development and a few are now being used successfully in cancer patient treatments. Nevertheless, adverse side effects and therapeutic resistance to RTK/PI3K inhibition remains problematic.

This review provides an overview of PTEN as a major regulator of RTK//PI3K/Akt activation and, in turn, discusses the regulation of PTEN by well characterised mechanisms, and more recently, by a novel mechanism involving regulation of PTEN by its pseudogene (PTENP1).

The clinical importance of PTEN inactivation in cancer and other diseases and the therapeutic potential of PTEN and PTENP1 modulation of the RKT/PI3K/Akt is discussed.

## PTEN sequence and structure

**Fig. 1:**
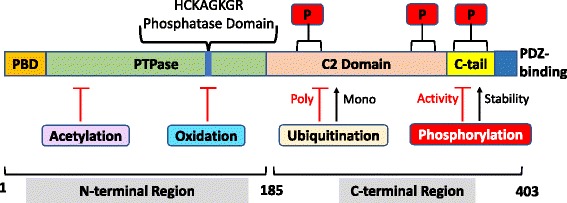
PTEN protein structure and sites of post-translational modification. PTEN is composed of 403 amino acids and is characterised by five functional domains: a phosphatidylinositol-4,5-bisphosphate (PIP2)-binding domain (PBD), a phosphatase domain containing the catalytic core, a C2 domain with putative ubiquitination sites, two PEST (proline, glutamic acid, serine, threonine) domains for degradation, and a PDZ interaction motif for protein-protein interactions. Post-translational regulation of PTEN occurs by ubiquitination (Ub) of Lys residues within the PBD and C2 domain, by oxidation, SUMOylation within the C2 domain, and acetylation on protein tyrosine phosphatase (PTPase) and PDZ-binding sites. Furthermore, PTEN is regulated by phosphorylation of specific serine and threonine residues within the C2 domain and C-tail terminal of PTEN (Modified from [14, 15])

The *PTEN* gene is encoded in 9 exons and has a 1212 nucleotide (nt) open reading frame. The gene encodes a polypeptide of 403 amino acids with a relative molecular mass of 47 kDa [8–12]. The PTEN protein consists of two major domains, the N-terminal phosphatase catalytic domain (residues 7–185) and a C-terminal domain (residues 186–351) [13–15] (Fig. 1). These two domains together form a minimal catalytic unit and comprise almost the entire protein, excluding only a very short N-terminal tail. The N-terminal phosphatase domain of PTEN contains a consensus PI (4,5) P2-binding motif. The C-terminal domain of PTEN contains the lipid binding C2 domain which confers affinity for phospholipid membranes in vitro. The C2 domain is believed to be required for the correct positioning of PTEN at the plasma membrane, the site of the lipid substrates of PTEN [13, 16–18]. The C-terminal tail of PTEN, consisting of the last 50 amino acids, also contains several phosphorylation sites that are critical for protein stability. Protein stability is dependent on the phosphorylation of S380, T382, and T383. Mutations within these sites reduce both the protein half-life and PTEN phosphatase activity [19]. Phosphorylation-defective mutants of PTEN have decreased protein stability and dephosphorylated PTEN is degraded by proteasome-mediated mechanisms [20, 21].

### PTEN cellular function and regulation of PTEN nuclear-cytoplasmic transport

Subcellular localisation of PTEN is crucial for its normal cellular function and its role as a tumour suppressor. PTEN translocates between the cytoplasm and nucleus of the cell and is known to have specific functions in both cellular compartments [6]. In the cytoplasm, PTEN interacts with its cytoplasmic targets to regulate cell proliferation, cell cycle progression, apoptosis, cell adhesion, migration and invasion. In the nucleus, PTEN plays a role in maintaining chromosomal stability and in DNA double strand break repair [6, 22], hence maintaining genome integrity. The mechanism(s) by which PTEN can translocate between the nucleus and cytoplasm of cells has not been completely characterised as PTEN does not appear to contain a traditional or consensus nuclear localisation signal (NLS), although putative NLS-like sequences have been identified [7].

The tumour suppressive role of cytoplasmic PTEN is through antagonism of PI3K/Akt signalling and the role of nuclear PTEN is to maintain chromosomal integrity and centromere stability. Mislocalisation of PTEN between the nucleus and the cytoplasm may lead to malignant growth, thus, the subcellular localisation of PTEN is closely regulated and several regulatory mechanisms have been identified. PTEN lacks a typical NLS, and monoubiquitination, active transport and passive diffusion has been identified as transport mechanisms for PTEN [23]. Monoubiquitination, catalysed by the ubiquitin-protein ligase, developmental downregulated-4-1 (NEDD4–1), enhances PTEN transport to the nucleus [24]. Nuclear pores are large enough to allow proteins of less than 60 kDa to pass through [25], thus making PTEN a perfect candidate for passage through the nuclear pore by diffusion. Ran (Ras-related nuclear protein) GTPase is able to actively transport PTEN into the nucleus [26]. A cytoplasmic localisation signal has been identified in the N-terminal domain of PTEN, spanning residues 19–25. Mutations in these residues (except residue 22) appear to increase nuclear localisation of PTEN, however the mechanism is not known [27]. Furthermore, mutations occurring at PTEN phosphorylation sites also appear to alter its nuclear-cytoplasmic localisation [26]. The stage of the cell cycle can also modulate the subcellular localisation of PTEN and the nuclear-cytoplasmic partitioning of PTEN can differentially regulate cell cycle progression and apoptosis [28]. The cell cycle dependent PTEN localisation can be regulated by Ca^2+^ mediated interaction with the major vault protein (MVP) [29]. Bipartite nuclear localisation sequences in PTEN are required for MVP mediated nuclear import and four such bipartite NLS have been identified and are responsible for MVP interaction [28].

### Regulation of PTEN abundance and activity

Since PTEN is involved in, and plays a central role in many cellular processes, the level of PTEN is tightly regulated by a number of cellular mechanisms which act at the transcriptional, post-transcriptional and post-translational levels and, as mentioned, small decreases in PTEN abundance or activity, may lead to tumourigenesis [2–5]. These regulatory mechanisms maintain the activity and abundance of PTEN at the required level under normal physiological conditions [30]. There are a number of well-established and documented regulatory mechanisms acting to modulate PTEN gene expression and protein abundance, stability and activity. However, more recently, PTEN regulation by the processed pseudogene of PTEN (PTENP1) is gaining much interest as an added level of complexity to the stringent regulation of PTEN expression.

In this section, we provide an overview of the well documented mechanisms of PTEN regulation, discuss the more recently defined mechanisms of PTEN regulation by small non-coding RNAs, microRNAs (miRNAs) and the exciting emerging field of pseudogene long non-coding RNAs (lncRNA). Importantly, we describe how the web of interactions between PTEN, PTEN-targeting miRNAs and the sense and antisense transcripts of the PTEN pseudogene, PTENP1, regulate RTK-dependent PI3K/Akt signalling [31–33].

#### Transcriptional regulation of PTEN

A number of transcription factors bind directly to the PTEN promoter to either activate or repress PTEN transcription. Such factors include the early growth response transcriptional factor 1 (EGR1), peroxisome proliferator-activated receptor gamma (PPARγ), [34, 35], activating transcription factor 2 (ATF2) [36] and the tumour suppressor, p53 [37]. p53 and PTEN share regulatory interactors and regulate each other in a feedback loop mechanism [38]. p53 upregulates PTEN transcription by binding to the functional p53 binding element upstream of the PTEN promoter [39]. PTEN is transcriptionally repressed by the zinc finger-like proteins SNAIL and SLUG, which are transcription factors competing with p53 for the PTEN promoter binding region [40]. Other transcription factors such as the polycomb group protein, CBF-1 and c-Jun, nuclear factor kappa-B and the antisense transcript of the PTEN pseudogene (PTENP1(AS)), also bind to the PTEN promoter and negatively regulate PTEN transcription [2, 15, 41, 42].

#### Post-transcriptional regulation of PTEN by miRNA

Recent advances in genomic technology have revolutionised the way we view cellular regulation, providing a greater appreciation and understanding of the complexity of non-coding genes and non-coding gene function(s). Once regarded as junk DNA, these non-coding genes have been shown to be critical in gene regulation and to play important roles in disease development and control. PTEN is regulated at the post-transcriptional level by miRNAs which are comprised of small ncRNAs approximately 14–24 nt in length [31]. These ncRNAs bind to their target messenger RNA (mRNA) at seed regions, known as miRNA recognition elements [43, 44], which are located within the 3’untranslated region (UTR) of the specific target mRNAs [45, 46]. Recent studies have revealed miRNA binding sites are also present in the coding regions, the 5’UTR region and even the promoter region of target mRNAs [46–48]. miRNA function is dependent on binding affinity with the target mRNA, therefore, binding of miRNAs can either lead to degradation of target through perfect complementary binding or inhibition of translation through imperfect binding [49, 50]. PTEN is known to be post-transcriptionally regulated by miRNAs binding within its 3’UTR, which results in blockage of translation, and a consequent decrease in PTEN abundance [51]. miRNAs commonly known to bind to, and repress PTEN include miR-17, miR-19, miR-21, miR-26, and miR-214 [32, 52, 53]. MiRNAs have been shown to possess functional roles in cancer development and progression [54], and a variety of oncogenic miRNAs (oncomirs) have recently been shown to bind specifically to PTEN transcripts, blocking PTEN translation, and to be cancer-type dependent. Overexpressing PTEN-specific miRNAs has the potential to enhance cancer progression, and specific PTEN-targeting oncomirs have been linked to hepatocellular carcinomas, prostate cancer, clear-cell renal carcinoma, breast cancer and endometrial cancer (Table 1). In 2010, a processed pseudogene of PTEN (PTENP1) was found to be transcribed to produce a transcript with high sequence similarity with the PTEN transcript. Further, this pseudogene transcript was ascribed a novel function by acting as a ‘decoy’ for miRNA binding of PTEN-targeting miRNAs, as discussed in more detail below [32].

**Table 1:** PTEN-targeting miRNAs identified in various cancer types

Cancer	microRNAs (miRNA, mIR)	References
Prostate	miR-17, miR-19, miR-21, miR-26 and miR-214	[32]
Hepatocellular	miR-17, miR-19b and miR-20a	[148]
Clear-cell renal	miR-21	[145]
Glioma	miR152	[152]
Breast	miR-106b and miR-93	[153]
Endometrial	miR-200a, miR-200b and miR-200C	[70, 127]

#### Pseudogenes and post-transcriptional regulation of PTEN by its pseudogene, PTENP1

The post-transcriptional regulation of PTEN by PTENP1 is a novel mechanism and sets a paradigm for regulation of cognate genes by their pseudogenes. This regulatory mechanism may provide new targets for cancer therapy or novel designs for cancer therapeutics.

##### Pseudogenes

**Fig. 2:**
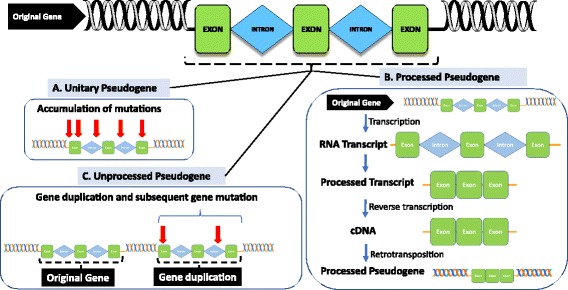
Pseudogene types shown to occur in the human genome. **a** Unitary pseudogenes are once functional gene sequences that have lost gene function due to the accumulation of mutations over time. **b** Non-processed pseudogenes are the result of direct duplication of existing genes, after which the duplicated version becomes inactivated due to the accumulation of mutations in sequences essential for gene expression. **c** Processed pseudogenes are the result of retrotransposition events. In this case, the mature mRNA transcript of a gene is reverse transcribed into a cDNA copy, which is then integrated into the genome of the organism. The site of integration of pseudogenes is random (Adapted from [61])

Knowledge of pseudogenes has existed for many years but their importance as post-transcriptional regulators of gene expression has only been recognised in recent years [55]. Since their initial identification, pseudogenes have been described in a wide range of species from bacteria [56], insects [57], plants [58] and animals [53]. Approximately 50% of transcribed pseudogenes in multicellular organisms exhibit evolutionarily conserved sequences across species, strongly suggesting a functional role for pseudogenes in humans and other organisms [59]. Pseudogenes are generally labelled as non-functional relatives of active genes that, over time, have lost their protein-coding ability, but share high sequence similarity with their cognate protein-coding genes. Despite the high sequence similarity, pseudogenes often contain nucleotide changes which prevent their translation to functional proteins. In the genome, pseudogenes are classified as either unitary pseudogenes, non-processed pseudogenes or processed pseudogenes. Unitary pseudogenes are those originating from native functional genes but which have lost their function due to mutations. Non-processed pseudogenes are a consequence of gene duplication while processed pseudogenes exist as an outcome of retrotransposition of mRNA transcripts [60, 61] (Fig. 2). Pseudogenes have generally been labelled as “junk” DNA as they are non-protein-coding sequences and their function, until recently, has been a mystery. Almost exact copies of their cognate genes, pseudogenes often harbour premature stop codons, deletions/insertions and frameshift mutations that cause their translation to non-functional proteins [62]. Because pseudogenes have lost the ability to produce full-length proteins, for many years, the assumption has been that they are non-functional, redundant, and evolutionary gene failures [63]. Whilst it has also been hypothesised for some time that antisense pseudogenes may bind to the sense parent gene transcript to regulate gene expression [64]. Although many pseudogenes are not transcribed due to inactive promoters, or their integration into silent regions of the genome, important roles have recently been highlighted through the discovery that some pseudogenes have the potential to regulate their protein-coding counterparts [32, 33, 61, 65]. Importantly, pseudogenes have recently been identified as modulators of disease processes, especially cancer [54, 66].

Pseudogenes are categorised as members of the LncRNA family, however some pseudogene transcripts have been shown to be processed into short interfering RNAs thereby regulating the coding genes through RNAi signalling [67]. As non-coding RNAs, pseudogenes offer an attractive control mechanism for gene regulation.

*The PTENP1 pseudogene* is evolutionarily conserved over many species, although the phylogenetic evolutionary history is complex (reviewed in [53]). During evolution, formation of the PTEN/PTENP1 gene families occurred through multiple gene duplication events. The human PTENP1 or ψPTEN is a processed pseudogene of PTEN located on chromosome 9p13.3. This pseudogene possesses extensive sequence identity to PTEN with only 18 nucleotide mismatches within the coding region [60, 68]. Sequence similarity between the 3′ untranslated regions (UTR) of PTEN and PTENP1 can be considered as occurring in two regions, the 5′ region with 95% sequence identity and the 3′ region with < 50% sequence identity [32]. Expression of PTENP1 leads to the production of three transcripts, two of which are antisense to PTEN (PTENP1 sense and antisense transcripts). One antisense transcript acts through binding chromatin remodelling complexes which alter H3K27me3 prevalence at the PTEN parental gene promoter [69]. The other antisense transcript is needed to stabilise the PTENP1 sense transcript, which lacks a poly-A tail.

##### The PTENP1 sense transcript acts as a ‘sponge’ to mop up PTEN-targeting microRNAs

**Fig. 3:**
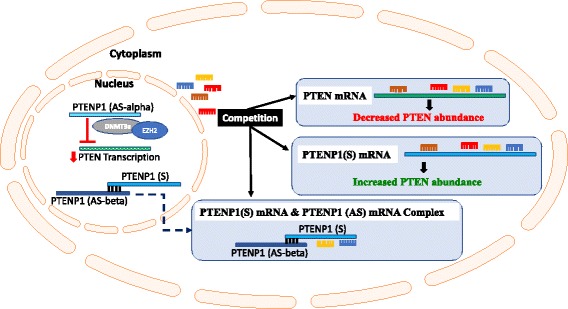
Regulation of PTEN by the sense and antisense transcripts of its processed pseudogene PTENP1: regulating the regulator of PI3K signalling. PTENP1 is transcribed into a sense and 2 antisense transcripts (a and b). In the cytoplasm, the sense transcript (PTENP1(S)) acts as competing endogenous RNA, competing with PTEN for the binding of PTEN-targeting miRNAs and thus freeing PTEN from miRNA-mediated repression and increasing PTEN cellular abundance. Of the 2 antisense PTENP1 transcripts, PTENP1(AS)α and PTENP1(AS)β produced, PTENP1(AS)α acts in the nucleus to negatively regulate PTEN transcription by recruiting chromatin-repressor proteins, the Enhancer of Zeste Homolog 2 and DNA methyltransferase 3a (EZH2) and DNA methyltransferase 3a (DNMT3a) to the PTEN promoter. Conversely, also in the cytoplasm, PTENP1(AS)β acts to stabilise the PTENP1(S) transcript through RNA-RNA interactions, as this the sense transcript lacks a poly(A) tail, and hence reinforces the miRNA ‘sponging’ activity of PTENP1(S) (modified from [42])

Many pseudogenes, while not being able to produce a functional protein are transcribed and act at the RNA level to regulate their coding counterparts, in part, by acting as decoys for microRNA binding [67]. Some pseudogenes display a tissue-specific pattern of action, and in their role as microRNA decoys, have the potential capacity to regulate oncogenes and tumour suppressor genes with tissue specificity (reviewed in [70]). Expression of the PTENP1 sense transcript is positively correlated with PTEN cellular abundance, consistent with a mechanism whereby the sense pseudogene transcript acts as a ‘sponge’ or ‘decoy’ for microRNAs that would otherwise bind the PTEN transcript and deactivate it (Fig. 3) [32]. Most interestingly, the 3’UTR sequences of PTEN and PTENP1 share common microRNA binding sites. PTENP1 was one of the first pseudogenes reported to be transcribed as a lncRNA and reported to function as ‘sponge’, or ‘decoy’, for miRNA binding to liberate PTEN from miRNA repression, hence restoring PTEN function [32]. Through binding of PTEN-targeting miRNAs, PTENP1 sense ultimately reduces the cellular concentration of these specific miRNAs. The PTEN/PTENP1 regulatory cycle is supported by experiments in which knockdown of PTENP1 results in decreased PTEN mRNA and protein levels [33].

Given that PTEN is a tumour suppressor gene, the PTENP1 pseudogene has been described as a tumour suppressor lncRNA pseudogene. Through its binding of PTEN-targeting miRNAs (Table 1), PTENP1 protects PTEN from miRNA binding and inhibition of PTEN translation [32]. Thus, PTENP1 acts as a repressor (molecular sponge) of the repressors (miRNAs) of PTEN function, and, in turn, regulates the regulator (PTEN) downstream of the RTK-dependent PI3K/Akt signalling pathway. These counteracting mechanisms illustrate the importance and complexity of the PTENP1 pseudogene as a lncRNA-mediator or regulator of PTEN expression and function.

#### Post-translational regulation of PTEN

**Fig. 4:**
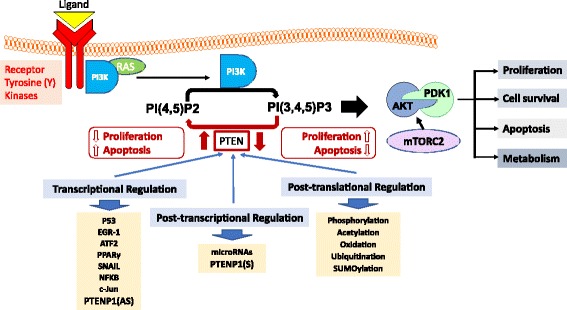
Regulation of PTEN, a major regulator of the PI3K/AKT signalling pathway. Growth factors bind receptor tyrosine kinases (RTKs) on the extracellular cell membrane, which leads to the recruitment and binding of PI3K (directly or through adaptor proteins) to its cytoplasmic domain through its regulatory subunit (P85). Activated PI3K phosphorylates of PI(4,5)P2 to PI(3,4,5)P3, which occurs through its catalytic subunit (P110). The serine/threonine kinases Akt and PDK1 are recruited to the membrane after binding to the pleckstrin homology (PH) domain of PI(3,4,5)P3. PDK1 and mTORC2 phosphorylate and activate Akt, which phosphorylates a number of downstream protein targets with the overall effect of enhancing cell proliferation, metabolism and survival whilst inhibiting apoptosis. PTEN is a major negative regulator of PI3K/Akt signalling through its phosphoinositide phosphatase activity which acts to directly antagonise Pi3K activity by dephosphorylating PI(3,4,5)P3 to PI(4,5)P2. PTEN abundance and activity is highly regulated through various complementary mechanisms working at the transcriptional, post-transcriptional and post-translational levels (modified from [14])

A number of post-translational mechanisms regulate PTEN activity and stability (half-life) and these include phosphorylation, oxidation, acetylation, ubiquitination and SUMOylation (Fig. 4).

##### Phosphorylation

PTEN catalytic activity may be modulated by phosphorylation of specific sites in the C2 and C-tail domains. Phosphorylation of particular serine and threonine residues (Ser380, Thr382, Thr383 and Ser385) in the C-terminal tail of PTEN, catalysed by the action of casein kinase 2 and glycogen synthase kinase 3ß [71], results in decreased phosphatase activity. The decreased phosphatase activity is associated with greater protein stability, or protein half-life, as a consequence of the generation of a closed-conformation through interaction of the C-terminal tail with the C2 domain [20]. Dephosphorylation results in a catalytically active open-conformation, but with decreased PTEN stability and cellular half-life [1, 2, 72].

##### Oxidation

PTEN oxidation by H_2_O_2_ facilitates disulphide bond formation between the catalytic Cys124 and Cys71 residues, resulting in a conformational change which alters the PTEN substrate binding site and leads to loss of PTEN phosphatase activity. PTEN oxidation is reversible through the action of thiol compounds, such as thioredoxin [73], and through PTEN interacting with peroxiredoxin-1 to prevent disulphide bond formation [74].

##### Acetylation

In response to growth factor stimulation, lysine acetyltransferase 2B (KAT2B), also known as PCAF, acetylates PTEN on lysine residues 125 and 128, which are located within the catalytic site of PTEN, and this results in the inactivation of PTEN phosphatase activity and the stimulation of PI3K signalling [75]. Another PTEN acetylation site is located in the PTEN PDZ-binding domain at Lys 402, which is driven by the CREB-binding protein. Acetylation of Lys 402 results in the negative regulation of PTEN activity [76]. Acetylation of PTEN may be reversed by the action of sirtuin 1, which restores the phosphatase activity of PTEN [77, 78].

##### Ubiquitination

Ubiquitination is a post-translational regulatory mechanism influencing the degradation [24] and compartmentalisation of PTEN [79]. The C-terminal tail and C2 domains of PTEN interact with each other to form a loop, which contains a major ubiquitination site (Lys 289). PTEN can be ubiquitinated by neural precursor cell expressing NEDD4–1 [79]. Polyubiquitination of PTEN results in decreased protein stability leading to PTEN degradation by proteasome mediated decay mechanisms [24], whereas monoubiquitination of PTEN on Lys13 and Lys289 promotes the nuclear transport of PTEN [24, 80].

##### SUMOylation

The attachment of small ubiquitin-related modifiers (SUMO) to proteins is also a post-translational regulatory mechanism of PTEN [81]. SUMOylation of PTEN on Lys266 facilitates the recruitment of PTEN to the plasma membrane [82], whereas, SUMOylation of PTEN on Lys254 partakes in controlling the nuclear localisation of PTEN [83]. SUMO proteins are related to ubiquitin [81]. SUMOylation of the Lys289 residue, a major monoubiquitination site for PTEN, results in PTEN nuclear localisation [24, 80]. Conversely, SUMOylation at Lys289 results in the recruitment of PTEN to the plasma membrane, implicating a possible competitive action for the modification of Lys293 [15, 81].

## The RTK/PI3K/Akt Signalling pathway and regulation by PTEN

RTKs are a family of transmembrane proteins with inherent phosphotyrosine kinase activity which remain inactive in the plasma membrane until activated by ligand. The RTK family consists of a number of sub-families, including the epidermal growth factor receptors (EGFRs), platelet derived growth factor receptors (PDGFRs), fibroblast growth factor receptors (FGFRs), vascular endothelial growth factor receptors (VEGFRs), insulin growth factor receptors (IGFRs), and hepatocyte growth factor receptors (HGFRs) [84–86]. Activation of the RTKs by their cognate growth factors, cytokines, hormones or other extracellular signalling molecules, triggers the activation of the PI3K signalling pathway. Tight regulation of cell proliferation by RTKs and their ligands is critical in cancer prevention [87].

The Class IA PI3Ks are heterodimeric enzymes consisting of a p110α /β /δ catalytic subunit and a p85 regulatory subunit and are directly activated by RTKs such as the insulin receptor (IR) and insulin-like growth factor receptor 1 (IGF-IR) [88, 89]. Class IB PI3K heterodimers consist of a p110γ catalytic subunit and a p101 regulatory subunit and are activated downstream of G-protein-coupled receptors (GPCRs). Class IA and IB PI3Ks are activated upon extracellular stimulation of RTKs or GPCRs, and, once activated, phosphorylate the D3-position of the inositol ring of phosphatidylinositol 4,5-bisphosphate (PtdIns(4,5)P2) to generate phosphatidylinositol 3,4,5-triphosphate (PtdIns(3,4,5)P3) at the plasma membrane. Both PtdIns(3,4,5)P3 and PtdIns(3,4)P2 facilitate the recruitment of pleckstrin homology-domain containing proteins, such as the serine/threonine kinase Akt [3–5], to the plasma membrane. Upon phosphoinositide binding, Akt is phosphorylated at Threonine-308 (Thr308) by phosphoinositide-dependent kinase 1 (PDK1) and at Serine-473 (Ser473) by the mammalian target of rapamycin complex 2 (mTORC2), leading to activation of its kinase activity and the subsequent phosphorylation of a number of target protein [90, 91].

PTEN is a well characterised negative regulator of PI3K-dependent Akt signalling. As a phosphoinositide phosphatase, PTEN acts as a direct antagonist of PI3K action through dephosphorylation of PtdIns(3,4,5)P3 at the D3-position of the inositol ring to form PtdIns(4,5)P2 [92–94]. Loss of PTEN, which occurs in many tumours, drives PI3K/Akt hyperactivation. The phosphoprotein phosphatase activity of PTEN has been linked to cancer signalling through dephosphorylation of protein targets such as focal adhesion kinase (FAK), insulin receptor substrate 1, c-SRC or PTEN itself [12, 95–97]. However, it is well established that, of the two activities of PTEN, it is the phosphoinositide phosphatase activity that plays the major tumour suppressor role [11, 98]. Of most importance, Akt hyperactivation resulting from the loss of PTEN lipid phosphatase function is the foremost oncogenic driving force in PTEN-deficient cancers. The protein phosphatase activity of PTEN is thought to be most important in the regulation of cell adhesion, cell migration, tumour metastasis and angiogenesis [99, 100]. Due to its importance in maintaining normal physiological functions in the cell, tight regulation of PTEN abundance and activity is essential for balancing cellular homeostasis (i.e. balancing cell proliferation and cell death).

### Clinical importance of PTEN mutations and PTEN deletions in cancer and other diseases

Germline mutations of PTEN have been linked to three autosomal dominant inherited cancer syndromes with overlapping features: Cowden Syndrome (CS), Bannayan Riley Ruvalcaba syndrome (BRRS), and Proteus syndrome (PS), all characterised by increased susceptibility to cancer [101]. These syndromes are notable for the presence of hamartomas, benign tumours in which differentiation is normal, but cells are highly disorganised. In these seemingly unrelated syndromes, PTEN germline mutations account for 80% of CS, 60% of BRRS, 20% of PS patients. A detailed comparative list of these PTEN mutations (CS, BRRS and PS), including their gene position, any associated amino acid changes and disease associations is provided in Table 3 in reference [102]. The features of CS include hamartomatous overgrowth of tissues and a predisposition to developing tumours of the breast, thyroid, endometrium and, in some instances, colon cancer [102]. An additional feature of CS is an increase in insulin sensitivity, which has been linked with PTEN haploinsufficiency-associated enhancement of PI3K/Akt signalling [103]. The majority of CS patients have macrocephaly and some patients also have autism spectrum disorders related to germline mutations of PTEN [104–108]. Over 80 different germline PTEN mutations have been identified, with specific mutations, including the R130X, Y178X nonsense and H93R, D252G, F241S missense mutations shown to be associated with the autism and macrocephaly characteristics and leading to the proposal that PTEN sequencing may allow genetic phenotyping and subsequent diagnosis of a subset of autistic patients [99].

BRRS is a rare hereditary autosomal dominant syndrome identified by developmental delays, megencephaly, speckled penis and lipomatosis [109]. There is some overlap in the germline mutations between CS and BRRS, however each syndrome has distinct PTEN germline mutations and, overall, distinct CS-associated mutations are located mainly in the 5′ exon-encoded region whereas BRRS distinct mutations occur mainly in the 3′-encoded C2 domain region [102].

The aetiology of PS is mostly considered as a germline mosaic mutation with features such as lipomas, overgrowth and benign neoplasms (hamartomas) [109]. At least three unique PS-associated PTEN mutations have been identified, W111R, C211X, M35 T and PS-like has a common mutation linked with both CS and BRRS [102].

Germline PTEN mutations associated with the hamartoma syndromes, as described above, are associated with patient predisposition to cancer. However, most cancers are associated with somatic alterations of PTEN being described in over 50% of all tumours of various types. In fact, PTEN is one of the most common targets for mutations in human sporadic cancers, with a mutational frequency rivalling that of p53 [1, 8, 9, 110, 111]. PTEN has been shown to be lost or inactivated by multiple mechanisms in a wide spectrum of human cancer types (Table 2). The spectrum of cancer-associated somatic mutations encompasses insertions, deletions, point mutations and epigenetic changes. Interestingly, in glioblastomas, loss of heterozygosity at the PTEN locus occurs in 60–80% of tumours and somatic mutations in 20–40% of such tumours [112]. Interestingly, haploinsufficiency or inactivation of a single PTEN allele has been shown to be sufficient for cancer development [3]. For example, key hereditary PTEN cancer-associated germline mutations and common somatic mutations with increased cancer risk have been identified in colorectal cancers [111, 113], breast cancers [114, 115], prostate cancers [116] and gliomas [117]. In tumours, PTEN is inactivated by various mechanisms, including not only mutations, but also deletions, transcriptional silencing through promoter hypermethylation, subcellular mislocalisation, and alterations of cellular stability and protein half-life as well as multiple mutations (reviewed in: [1, 110]. Loss of PTEN is commonly observed in glioblastoma, thyroid, breast, endometrial, ovarian, prostate, colorectal cancers, and melanoma [8, 9, 110, 111].

**Table 2:** PTEN status of various cancer types as adapted from reference [133]

Cancer type	PTEN status	References
Head and neck cancer	- Decreased PTEN expression in 30% of patients	[154]
Glioblastoma	- Loss of heterozygosity of PTEN in 60%–80% of patients	[155]
	- PTEN mutations in up to 40% of patients	
Breast cancer	- PTEN mutations in 3% of patients	[156, 157]
	- Loss of PTEN protein in 30% of patients	
Ovarian cancer	- Loss of heterozygosity of PTEN in 45% of endometrioid carcinoma of the ovary	[158]
Non-small cell lung cancers	- Loss of PTEN protein expression in 75% of patients	[159]
Endometrial cancer	- Mutation or reduction of heterozygosity in 55% of patients	[160]
Colorectal cancer	- Loss of PTEN protein expression in 20%-40% of patients	[161]
Prostate cancer	- PTEN mutations in 15% of primary tumours, 20% of localised tumours and 50% of hormone-refractory cancer patients	[162]

As cellular PTEN concentration strongly influences cancer development, and subsequent cancer severity [5], maintenance and control of cellular PTEN levels is critical for preventing oncogenesis. For example, loss of PTEN is associated with progression of prostate cancers from the androgen-dependent to the more aggressive androgen-independent phenotypes, resistance to chemo- and radiation therapies, tumour metastasis, recurrence post-surgery, and significant overall poor prognosis for patients [118].

PTEN abnormalities extend far beyond cancer related diseases. Changes in PTEN cellular levels, and related cellular compartmentation, have also been implicated in prominent diseases such as diabetes and neurological disorders including Parkinson’s disease and Alzheimer’s disease [63, 105, 107, 119–121]. Inappropriate activation of the PI3K/Akt pathway, consequent to PTEN loss through gene deletions or mutations, especially those affecting the active site residues, has been suggested as a mechanism involved in adverse neuropsychiatric cell signalling [58]. Also suggested by Kitagishi and Matsuda [58] is the potential of targeting the PI3K signalling pathway in the treatment of neurological impairment such as that seen in Parkinson’s disease. PTEN haploinsufficiency also underlies profound insulin sensitivity resulting in predisposition to obesity and diabetes type II, as well as cancer [122]. A common PTEN variant, rs1102614, has been linked to peripheral insulin resistance and development of Type II diabetes [123].

Here, we have highlighted some of the more prominent diseases associated with PTEN mutations, however as more PTEN genetic data emerges, the importance of PTEN as a major checkpoint and regulator of disease will undoubtedly increase.

#### PTENP1 regulators in disease

Given their potential regulatory role in normal cellular function, it is not unconceivable that specific changes in pseudogene expression occur and contribute to disease progression. Examples of changing dynamics in pseudogene expression have been shown in some cancers [124, 125] and in diabetes [126], two major diseases of the developed world. The PTENP1 pseudogene, as a key player in PTEN regulation, has the potential to strongly influence tumour development and progression. Fluctuating levels of PTEN/PTENP1 are often correlated in prostate cancer samples and deletion of PTENP1 occurs frequently in some sporadic cancers such as endometrial, colon and prostate cancers, attributing a tumour suppressor function to PTENP1, that is independent of its regulation of PTEN [32, 127, 128]. A further example of the action of the PTENP1 antisense transcript is PTENP1(as) has been shown to alter doxorubicin sensitivity in cancer cells, a clinically actionable phenotype [69].

### Cancer therapeutic potential of PTEN: Modulating RTK-dependent PI3K/Akt overactivation

Aberrations in the PI3K pathway are common to many cancer types and targeting the RTK/PI3K/Akt pathway continues to provide key opportunities for therapeutic intervention. Overactivation of the RTK pathway is endemic in cancer progression and tight downstream regulation of this pathway is enforced in the cell at many levels. The employment of RTK inhibitors as therapeutic agents has been a major breakthrough in the treatment of cancers such as melanoma (BCR-ABL, KIT, PDGFR), breast cancer (Herceptin 2: HER2), colorectal cancer (EGFR, VEGF) and non-small cell lung cancer (EGFR) [129], and, to-date, the Food and Drug Administration (FDA) have approved 26 kinase inhibitors for cancer treatment, of which 8 are TK inhibitors [130]. However, intrinsic (primary) and acquired (secondary) resistance to conventional drug regimes is the major challenge to overcome in cancer therapeutics. Each step in the RTK cascade is a potential cancer target. Understanding the signalling pathways associated with RTK signalling networks and targeting intermediates in the PI3K/PTEN pathway may be a step forward in diagnostics/prognostics and allow translatable approaches in new therapeutic designs to potentially overcome drug resistance.

Specific PI3K inhibitors are proving to be promising cancer targets though few have made it into successful clinical outcomes. One such inhibitor identified is the PI3Kδ inhibitor Idelalisib, currently approved for use in patients with chronic lymphocytic leukaemia, small lymphocytic lymphoma and follicular lymphoma [129]. There are a number of PI3K targeting drugs currently under development, and in various stages of clinical trials (phase II-III) from pan-class 1 PI3K inhibitors such as buparlisib (BMK120), Copanlisib (BAY80–6946) and pictilisib (GDC-094), which target all four PI3K isoforms, to PI3K isoform-specific inhibitors such as IPI-145 and Alpelisib [116]. PI3K inhibitors, BAY80–6946 (Copanlisib), GDC0032 and IPI145, which target PI3Kα, PI3Kβ, PI3Kδ and PI3Kγ, are undergoing phase II–III trials for treatment of lymphoma, breast/uterine cancer and lymphocytic leukemia/lymphoma respectively [131, 132]. Furthermore, a number of the PI3K isoform-specific inhibitors are in stage I or II of clinical trial, including, but not limited to, NVP-BYL719 or Alpelisib (targeting PI3Kα, PI3Kβ and PI3Kγ), INK1117 or MLN1117, SAR260301, KIN-193 or AZD6482, GS-9820 (all targeting PI3Kα, PI3Kβ, PI3Kδ and PI3Kγ), GSK2636771 (targeting PI3Kβ) and AMG319 (targeting PI3Kδ) [132–135]. Other inhibitors currently in preclinical trial are described in detail in [132–135].

Clinical trials with AKT inhibitors have shown limited clinical success, and miltefosine is currently the only approved therapy as a typical treatment for cutaneous breast cancer [136].

Targeting PTEN *per se*, as a cancer therapeutic strategy, is very problematic given its key role in cell regulation and proliferation and changes in PTEN expression can trigger profound biological effects. Therapeutic approaches to increase PTEN levels have anti-cancer benefits however increasing PTEN has a positive influence in tissue regeneration [137].

On the one hand, increasing functional dose/ levels of PTEN has been shown to promote its tumour suppressor activity, thus making PTEN a good candidate for cancer treatment. Insertion of PTEN protein in PTEN null prostate cancer cells (PC-3) [138] induced apoptosis and regression of PTEN-null xenograft tumors in mice [139]. Interestingly, introduction of additional exogenous PTEN expression by generating PTEN-transgenic mice, or “Super-PTEN” mice, reduced cancer susceptibility by altering cellular cells’ metabolic pathway, negatively impacting the ‘Warburg effect’, a metabolic feature of tumour cells [140]. Notably, these “Super-PTEN” mice, showed reduced body size and a decrease in cell number with a positive healthy metabolism [141]. Based on these findings, pharmaceutical delivery of functional dosage of PTEN through PTEN protein delivery, inhibition of PTEN-targeting miRNAs, and PTEN gene editing would benefit cancer patients.

On the other hand, as mentioned, decrease of functional PTEN dose increases cell growth and proliferation, which is shown to be useful in regenerative medicine for Alzheimer’s disease and ischemia however decreasing functional PTEN dose has the potential for tumourigenicity [5]. Conditional PTEN deletion leads to mTOR activation and stimulates and promotes axon regeneration as demonstrated in crush injuries in corticospinal neurons [142]. Cardiac specific deletion of the PTEN gene in a mouse model protected cardiac myocytes after cardiac ischemia/reperfusion injury by inhibiting anti-apoptotic signals [143]. In a recent study on cellular and animal models of Alzheimer's disease, it was illustrated that inhibition of PTEN saved the normal synaptic function and cognition [144]. Modulation strategies used for functional PTEN reduction include, direct protein inhibition through inhibition of PTEN phosphatase activity or inhibition of PTEN by protein-protein interaction, targeting of PTEN mRNA to reduce PTEN protein translation and gene editing through new technologies such as C2c2, CRISPR/Cas 9, or Cpf1.

Understanding the roles of pseudogenes, such as PTENP1, which has come to the forefront as a modulator of PTEN, and regulatory functions thereof, may improve our current knowledge of tumour biology, providing a new perspective for the discovery of candidate drugs as opportunistic therapies as well as future biomarkers. There is accumulating evidence that lncRNA PTENP1 possesses a tumour suppressive role in several cancers and has been downregulated or deleted in numerous cancers such as prostate, gastric carcinoma, clear-cell renal carcinomas, lung cancer, melanoma and colon cancer [32, 128, 145–147]. The overexpression of PTENP1 in cell lines and in in vivo studies has been shown to regulate cell proliferation, reduce tumour growth, invasion, metastasis and apoptosis [147–151], further solidifying the importance of PTENP1 in regulating the biology of a cell by acting as a tumour suppressor, independent of PTEN.

Reduction in PTENP1 expression has been presented in numerous cancer studies and has been predicted to be a promising candidate as a future prognostic biomarker [32, 128, 145–147, 151]. A personalised medicine approach is possible in the distant future, however, before this can become a reality, a complete understanding of the multiple layers and complexity of the regulation of the regulator of the RKT-dependent PI3K/AKT pathway, PTEN, and its pseudogene (PTENP1), the regulator of PTEN, and its antisense transcripts, needs to be further investigated and understood. One of the major considerations in modulating PTEN/PTENP1 in cancer therapy is the majority of cancers are age related. Many diseases, which would not benefit from increased PTEN, such as reduction in cognitive functions, including Alzheimer's, are more prevalent with aging.

## Summary and conclusion

PTEN is dysregulated in many human cancers, and recent studies highlight the complexity of regulation of PTEN expression. Ablation of PTEN can drive oncogenic PI3K signalling, leading to diverse phenotypic outcomes. The relative expression levels of PTEN, and its sense and antisense pseudogene transcripts may mediate this distinction whereby different levels of these transcripts are expressed in different tumour types or tumours of variable stages and histological grades. PTEN and its pseudogene transcripts have specific subcellular localisations and thus it is conceivable that compartmentalisation of PTEN, PTENP1(s) and PTENP1(as) may contribute to their observed downstream function. Further investigation of PTEN and PTENP1 transcript dysregulation within different cancer types may help define the highly dynamic and complex regulatory role the PTEN pseudogene lncRNAs play in tumourigenesis and determine whether miRNA-based treatments, or other alternative approaches will be effective cancer therapeutic strategies. Here, we have highlighted a framework for identification of intermediaries and downstream modulators in the RTK-dependent PI3K/Akt pathway which can be targeted for diagnosis, prognosis and treatment of cancer. The challenge is now to determine the pathways to intrinsic and acquired resistance and to identify potential candidate cancer-related intermediaries, such as the PTEN pseudogene, as potential biomarkers and therapeutic targets.

*In conclusion,* an in-depth understanding of novel mechanisms of RTK/PI3K/Akt regulation may present new cancer therapeutic targets and opportunities through the targeting of key regulators of cell signalling downstream of RTKs, such as the PTEN/PTENP1 rheostat.

## Abbreviations

PPARγ: Peroxisome proliferator-activated receptor gamma
AKT: Protein kinase B
ATF2: Activating transcription factor 2
BRRS: Bannayan Riley Ruvalcaba
CS: Cowden Syndrome
EGFR: epidermal growth factor receptor
EGR1: early growth response transcriptional factor 1
FDA: Food and Drug Administration
FGFR: Fibroblast growth factor receptor
GPCR: G-protein-coupled receptor
HGFR: Hepatocyte growth factor receptor
IGFR: Insulin growth factor receptor
IR: Insulin receptor
microRNA: miRNA, miR
mRNA: messenger RNA
MVP: Major vault protein
ncRNA: Non-coding RNA
NEDD4–1: Neural precursor cell expressed developmental downregulated-4-1
NLS: Nuclear localisation signal
Nt: Nucleotide
PBD: PIP2-binding domain
PDGFR: Platelet derived growth factor receptor
PDK1: Phosphoinositide-dependent kinase 1
PEST: Proline, glutamic acid, serine, threonine
PI3K: Phosphoinositide 3-kinase
PS: Proteus Syndrome
PtdIns(3,4,5)P3: Phosphatidylinositol 3,4,5-trisphosphate
PtdIns(4,5)P2: Phosphatidylinositol-4,5-bisphosphate
PTEN: Phosphatase and tensin homologue
PTENP1: PTEN pseudogene
PTENP1(AS): PTENP1 antisense transcript
PTPase: Protein tyrosine phosphatase
Ran: Ras-related nuclear protein
RKT: receptor tyrosine kinase
RNAi: RNA interference
SUMO: Small ubiquitin-related modifiers (SUMO)
Ub: Ubiquitination
UTR: Untranslated region
VEGFR: Vascular endothelial growth factor receptor
